# Peer Review of “Toward Human Digital Twins for Cybersecurity Simulations on the Metaverse: Ontological and Network Science Approach”

**DOI:** 10.2196/38583

**Published:** 2022-04-20

**Authors:** Jorge Ropero

**Affiliations:** 1 Universidad de Sevilla Sevilla Spain

**Keywords:** human behavior modeling, cognitive twins, human digital twins, cybersecurity, cognitive systems, digital twins, Metaverse, artificial intelligence

*This is a peer-review report submitted for the paper “Toward Human Digital Twins for Cybersecurity Simulations on the Metaverse: Ontological and Network Science Approach.”*

## Round 1 Review

### General comments

This paper [1] deals with the use of Digital Twins in Cybersecurity, proposing a conceptual framework. The starting point is interesting, but I have found the following issues.

### Specific Comments

#### Major Comments

1. The author states that this paper proposes an application of Digital Twins (DT) and Human Digital Twins (HDT) for the first time. This is not exact, as, in the last 2 years, there have been some approaches to the use of DT in cybersecurity.

The author should include some of these ideas in the literature review. Some examples are listed below.

Lou X, Guo Y, Gao Y, Waedt K, Parekh M. An idea of using Digital Twin to perform the functional safety and cybersecurity analysis. *INFORMATIK 2019: 50 Jahre Gesellschaft für Informatik–Informatik für Gesellschaft (Workshop-Beiträge)*. Gesellschaft für Informatik eV. 2019;295:283-294.

Scheibmeir J, Malaiya YK. Multi-model security and social media analytics of the digital twin. ASTEJ. 2020;5(6):323-330.

Atalay M, Angin P. A digital twins approach to smart grid security testing and standardization. IEEE International Workshop on Metrology for Industry 4.0 & IoT 2020 Jun 3; Rome, Italy. pp 435-440.

Pokhrel A, Katta V, Colomo-Palacios R. Digital twin for cybersecurity incident prediction: A multivocal literature review. Proceedings of the IEEE/ACM 42nd International Conference on Software Engineering Workshops 2020 Jun 27; Seoul, Republic of Korea. pp 671-678.

Saad A, Faddel S, Youssef T, Mohammed OA. On the implementation of IoT-based digital twin for networked microgrids resiliency against cyber attacks. IEEE transactions on smart grid. 2020 Jun 9;11(6):5138-5150.

Olivares-Rojas JC, Reyes-Archundia E, Gutiérrez-Gnecchi JA, Molina-Moreno I, Cerda-Jacobo J, Méndez-Patiño A. Towards Cybersecurity of the Smart Grid using Digital Twins. IEEE Internet Computing. 2021 Mar 3.

2. In the literature review, the author should add a definition of DT and HDT, how HDT surges from the concept of DT, a comparison between both techniques, and finally a list of the main uses of DT and HDT.

3. In the literature review, the author claims that there is no grounded vision of the power of DT and HDT. In addition to the fact that, as I mentioned before, there are already applications of DT to cybersecurity, nothing is mentioned about proactive cyber defense existing techniques. What can DT and HDT add to the existing techniques?

Husák M, Bartoš V, Sokol P, Gajdoš A. Predictive methods in cyber defense: Current experience and research challenges. Future Generation Computer Systems. 2021 Feb 1;115:517-530.

4. The author states that the framework targets the cognitive process of a malicious actor as an HDT within a DT system. What is the purpose of this? The author must explain why these decisions were made.

5. Regarding Table 1, how was the total score calculated? There should be a description of every item. How was the score of every item calculated? An explanation is necessary.

6. Related to the above, it is good to have all the information in GitHub, but, at least a brief and clear description of the obtention of cybersecurity-related behavioral theories, and another description of the ontology should be provided in the manuscript or in a Multimedia Appendix.

7. An explanation of Figure 1 is needed.

8. Without a clear description, the rest of the paper, although interesting, is difficult to follow.

9. In broad terms, I understand the goal of the ontology, but it is so abstract that it is difficult for me how to apply it to proactive cyber defense. Some examples would be welcome.

10. Last, a general comment: this is the Journal of Medical Internet Research. Though other topics are welcome, and it is clear that security is capital in the medical field, some particular comments about cybersecurity in the medical field would be desirable.

#### Minor Comments

11. In the introduction, the author states that “incredibly,” HDT offers the capability of running large-scale simulations. Why “incredibly”?

12. In the introduction, the author claims that “Analyzing the Cybonto ontology informed the Cybonto conceptual framework.” I do not understand this sentence.

13. The author defines the in-group environment acronym as IGE, but it appears as IEG in the rest of the paper.

## Round 2 Review

### General Comments

Though all the comments have not been directly addressed by the author, the author has considered some aspects (many of them related to the state of the art) to be beyond the scope of the paper and has structured the article in a more ordered way. Thus, the paper is much easier to understand.

The inclusion of the Discussion section is key to see the applicability of the framework.

### Specific Comments

#### Major Comments

1. In Table 2, what are PR, EC, BC, and DC?
